# Supplementary material for: Splice-Junction-Based Mapping of Alternative Isoforms in the Human Proteome
Source: Cell Rep. Author manuscript; Available in PMC 2020 Jan 15. (PMC6961840; doi:10.1016/j.celrep.2019.11.026)
Supplement: 2 [file NIHMS1546469-supplement-2.zip › DF1/PXD009737/TestisGluC/TestisGluC_5_SYPL1_TSLHSPSNTSAPHSQGGIPPPTGL.pdf]

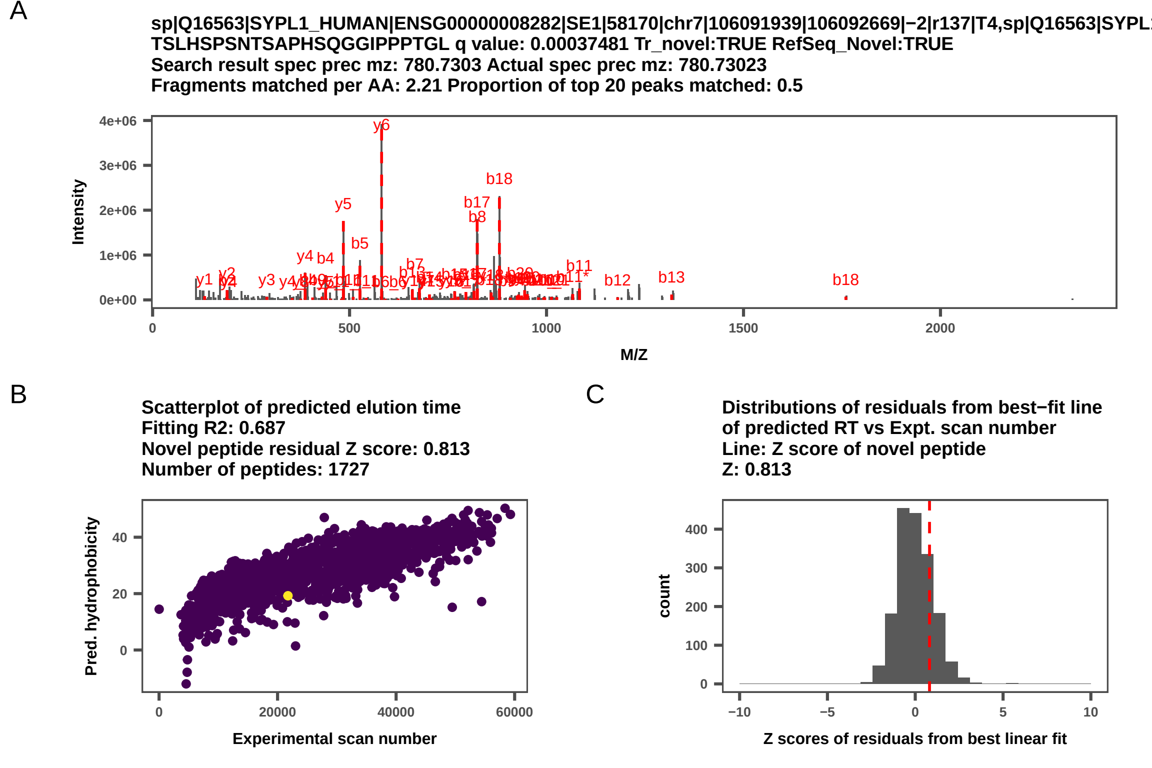
A
sp|Q16563|SYPL1_HUMAN|ENSG00000008282|SE1|58170|chr7|106091939|106092669|−2|r137|T4,sp|Q16563|SYPL
TSLHSPSNTSAPHSQGGIPPPTGL q value: 0.00037481 Tr_novel:TRUE RefSeq_Novel:TRUE
Search result spec prec mz: 780.7303 Actual spec prec mz: 780.73023
Fragments matched per AA: 2.21 Proportion of top 20 peaks matched: 0.5
Intensity
4e+06
3e+06
2e+06
1e+06
0e+00
y6
b18
y5
b17
b8
b5
y4
b4
b7
b11
b12
b13
y1
y2
y3
0
500
1000
1500
2000
M/Z
B
Scatterplot of predicted elution time
Fitting R2: 0.687
Novel peptide residual Z score: 0.813
Number of peptides: 1727
Pred. hydrophobicity
40
20
0
0
20000
40000
60000
Experimental scan number
C
Distributions of residuals from best−fit line
of predicted RT vs Expt. scan number
Line: Z score of novel peptide
Z: 0.813
count
400
300
200
100
0
−10
−5
0
5
10
Z scores of residuals from best linear fit
